# Left ventricular assist device for end-stage heart failure: results of the first LVAD destination program in the Netherlands

**DOI:** 10.1007/s12471-014-0602-4

**Published:** 2014-10-02

**Authors:** M. L. A. Haeck, S. L. M. A. Beeres, U. Höke, M. Palmen, L. E. Couperus, V. Delgado, E. A. Logeman, J. J. Maas, R. J. M. Klautz, M. J. Schalij, H. F. Verwey

**Affiliations:** 1 Department of Cardiology, Leiden University Medical Center, Albinusdreef 2, 2333 ZA Leiden, the Netherlands; 2 Department of Cardiothoracic Surgery, Leiden University Medical Center, Leiden, the Netherlands; 3 Department of Anesthesiology, Leiden University Medical Center, Leiden, the Netherlands; 4 Department of Intensive Care Medicine, Leiden University Medical Center, Leiden, the Netherlands

**Keywords:** End-stage heart failure, Left ventricular assist device, Destination therapy

## Abstract

**Purpose:**

Mechanical circulatory support with a continuous-flow left ventricular assist device (LVAD) may be a valuable treatment in end-stage heart failure patients for an extended period of time. The purpose of this study was to evaluate the safety and efficacy of implantation of a continuous-flow LVAD in end-stage heart failure patients within the first destination program in the Netherlands.

**Methods:**

A third-generation LVAD was implanted in 16 heart failure patients (age 61 ± 8; 81 % male; left ventricular ejection fraction 20 ± 6 %) as destination therapy. All patients were ineligible for heart transplant. At baseline, 3 and 6 months, New York Heart Association (NYHA) functional class, quality-of-life and exercise capacity were assessed. Clinical adverse events were registered.

**Results:**

Survival at 30 days and 6 months was 88 and 75 %, respectively. In the postoperative phase, 6 (38 %) patients required continuous veno-venous haemofiltration for renal failure and 2 (13 %) patients required extracorporeal membrane oxygenation because of severe right ventricular failure. During follow-up, NYHA functional class and quality-of-life improved from 3.7 ± 0.1 to 2.3 ± 0.1 and 57 ± 5 to 23 ± 3 at 6 months (*P* < 0.001), respectively. The 6 min walking distance improved from 168 ± 42 m to 291 ± 29 m at 6 months (*P* = 0.001).

**Conclusion:**

Continuous-flow LVAD therapy is a promising treatment for patients with end-stage heart failure ineligible for heart transplant.

## Introduction

End-stage heart failure is a challenging syndrome with an increasing incidence and prevalence worldwide. Despite advances in medical therapy and cardiac surgery, heart failure frequently progresses and overall survival and quality-of-life is poor [1–3]. The first-choice treatment option for end-stage heart failure remains heart transplant [4]. However, access to this therapy is limited due to scarcity of donor hearts. Mechanical circulatory support with continuous-flow left ventricular assist device (LVAD) is an emerging technique that may support end-stage heart failure patients for an extended time period and offers an alternative treatment option [5]. Traditionally, LVADs were intended as bridge to transplant, when patients receive LVAD for a relatively short period while on the waiting list. Improvements in the design of the LVADs led to extended duration of mechanical support and eventually to the use of LVADs as destination therapy, when recovery cannot be expected and transplant is not feasible [6].

Several studies have demonstrated improved survival, functional capacity and quality-of-life in patients receiving LVAD therapy compared with patients on optimal medical treatment [7–11]. However, there is still concern about the adverse events of LVAD therapy such as infections, thromboembolic events and mechanical failures [7, 8, 11]. Although the use of LVADs has been approved as bridge to transplant in the Netherlands, there is no experience to date with LVADs in end-stage heart failure patients as destination therapy. Considering the implications LVAD destination therapy may have on the growing population of heart failure patients, further research on this topic is needed. The purpose was to evaluate the safety and efficacy of implantation of continuous-flow LVAD as destination therapy in end-stage heart failure patients who were not candidates for transplant.

## Methods

### Patient selection

The study population consisted of the first 16 consecutive patients with end-stage heart failure undergoing implantation of an LVAD as destination therapy at the Leiden University Medical Center. By definition, all patients had New York Heart Association (NYHA) class IIIb or IV heart failure symptoms despite optimal medical therapy. A multi-disciplinary cardiothoracic team determined the ineligibility for guideline-recommended therapy (including revascularisation, cardiac resynchronisation therapy and heart failure surgery) after analysis of the patient according to the previously described MISSION! Heart Failure protocol [12]. If patients were not considered candidates for transplant, further screening was performed according to the MISSION! LVAD protocol.

**Fig. 1:**
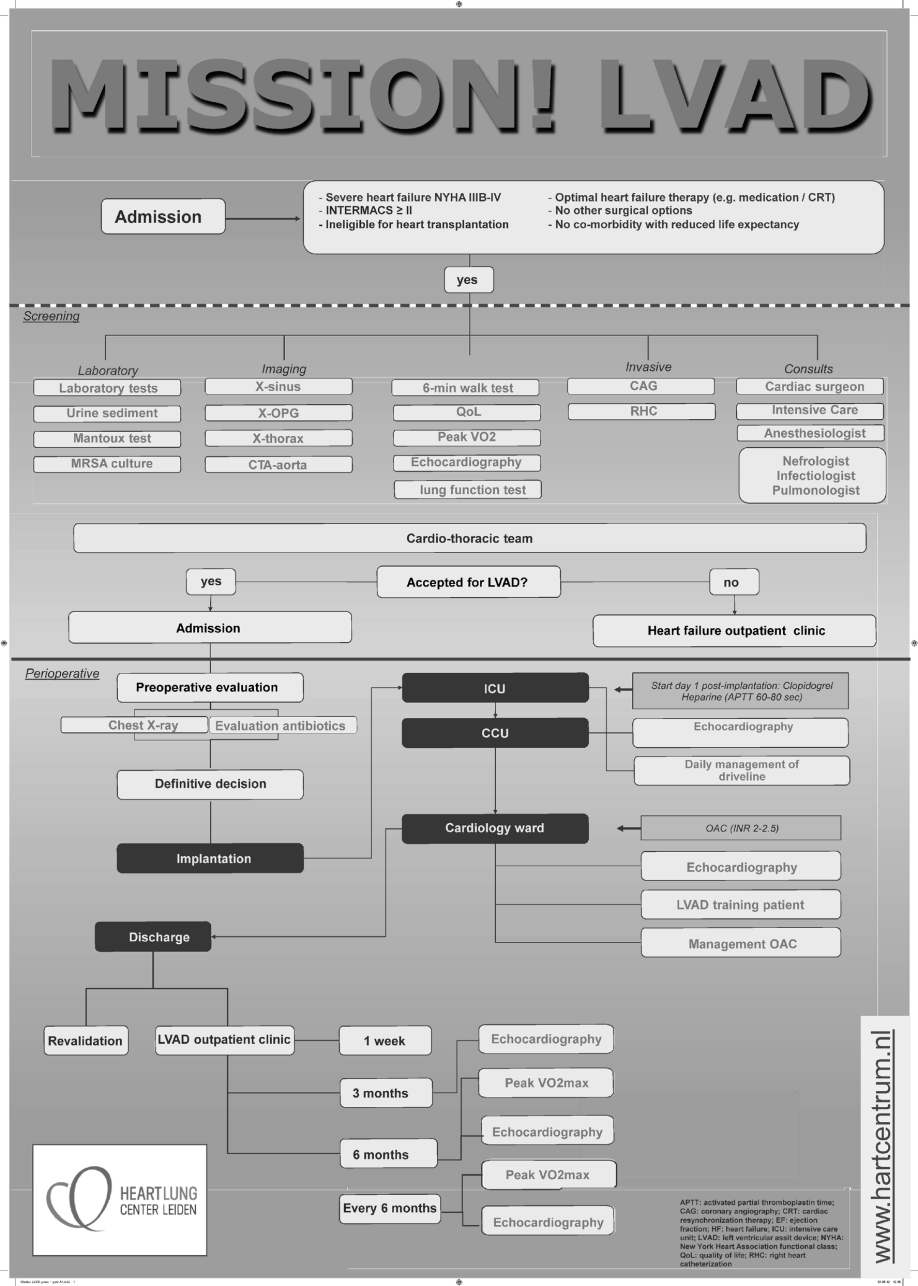
Flow chart of left ventricular assist device destination program

The MISSION! LVAD protocol comprises an extensive screening to provide more insight into the clinical status of the patient (Fig. 1). In particular, the Interagency Registry for Mechanically Assisted Circulatory Support (INTERMACS) level is determined, which represents a reclassification of NYHA class IIIb-IV heart failure in order to improve selection of patients for LVAD therapy [13]. Furthermore, quality-of-life is determined by the Minnesota Living with Heart Failure questionnaire, exercise capacity is assessed by a 6 min walk test and, if feasible, maximal oxygen uptake (VO_2_ max) is measured during bicycle exercise testing. An essential part of the screening is to detect potential contraindications for long-term LVAD support including non-cardiac morbidity limiting life expectancy to <2 years, severe right ventricular dysfunction, active systemic infection, significant renal dysfunction (GFR <30 ml/min) and contraindications for chronic antithrombotic therapy. Also, INTERMACS level 1 was considered a contraindication for LVAD implantation since previous studies showed an increased risk of major adverse events in these patients [14, 15]. The decision to accept or reject patients for LVAD implantation was made in a multi-disciplinary team including cardiologists, thoracic surgeons, anaesthesiologists and intensive care specialists.

### LVAD implantation

All patients received a HeartWare VAD (HeartWare Inc, Framingham, MA) [16]. Implantation of the LVAD was performed according to the HeartWare instructions for use. Pump speed was optimised peroperatively and in the intensive care unit (ICU) using Swan-Ganz catheter measurements and echocardiography. All patients were treated with inhaled nitric oxide during implantation and the postoperative phase at the ICU. After implantation, an antithrombotic regimen was initiated with heparin and clopidogrel. After haemodynamic stabilisation, heparin was replaced by oral anticoagulation and conventional oral heart failure medication was re-introduced.

### Follow-up

During follow-up, adverse events including death, stroke, renal failure (defined as the need for continuous veno-venous haemofiltration (CVVH)), severe right ventricular failure (defined as the need for extracorporeal membrane oxygenation (ECMO)), LVAD-related infections (driveline exit site and pump pocket) and device failure were recorded. The cause of death was determined by examination of the medical reports. After hospital discharge, patients were followed at the LVAD outpatient clinic as per protocol. At 3 and 6 months, laboratory measures, NYHA functional class and quality-of-life were re-assessed. Exercise capacity was determined with the 6 min walk test and with the VO_2_ max uptake.

### Statistical analysis

Continuous data are presented as mean ± standard deviation or mean ± standard error of the mean, as appropriate, and categorical data are presented as frequencies and percentages. Changes in NYHA functional class, quality-of-life score, exercise capacity and laboratory tests were evaluated using linear mixed model analyses. A *P*-value <0.05 was considered statistically significant. All data were analysed using the software SPSS (SPSS17.0, SPSS Inc, Chicago, USA).

## Results

The baseline characteristics are summarised in Table 1. Mean age was 61 ± 8 years and mean left ventricular ejection fraction was 20 ± 6 %. The main reasons for rejection for heart transplant were malignancy in the medical history, severe renal dysfunction, and irreversible pulmonary hypertension. At baseline, 5 (31 %) patients had NYHA class IIIb symptoms and 11 (69 %) patients had NYHA class IV. The number of days that patients had been hospitalised for heart failure in the year prior to LVAD implantation was 54 ± 55 days. According to the INTERMACS level, 4 (25 %) patients were in level 5 (exercise intolerant), 3 (19 %) patients were in level 4 (recurrent advanced heart failure), 7 (44 %) patients were in level 3 (stable but inotrope-dependent) and 2 (13 %) patients were in level 1 (critical cardiogenic shock). These last two patients were in level 2 and 3 at the time of acceptance; however the clinical situation deteriorated in the days before the operation requiring implantation of an Impella (Abiomed, Danvers, MA) device and an intra-aortic balloon pump, respectively.

**Table 1:** Baseline characteristics of patient population (*n* = 16)

Age (years)	61 ± 8
Male gender	13 (81 %)
BSA (m^2^)	2.00 ± 0.17
NYHA functional class	
IIIb	5 (31 %)
IV	11 (69 %)
INTERMACS level at the moment of surgery	
1	2 (13 %)
2	-
3	7 (44 %)
4	3 (19 %)
5	4 (25 %)
LVEF (%)	20 ± 6
Ischaemic aetiology	13 (81 %)
Cardiac index (l/min/m^2^)	2.18 ± 0.43
RVSP (mmHg)	46 ± 14
MPAP (mmHg)	32 ± 9
Diabetes mellitus	4 (25 %)
Cardiac resynchronisation therapy	12 (75 %)
Previous thoracic surgery	9 (56 %)
Inotrope-dependent heart failure last 6 months	11 (69 %)
Intra-aortic balloon pump or Impella	2 (13 %)
No. of hospital days one year pre-implantation	54 ± 55

*BSA* body surface area; *LVEF* left ventricular ejection fraction; *MPAP* mean pulmonary artery pressure; *NYHA class* New York Heart Association class; *RVSP* right ventricular systolic pressure

### Perioperative data

The LVAD was successfully implanted in all 16 patients. The mean operating time was 443 ± 146 min and mean perfusion time was 179 ± 66 min. The concomitant surgical procedures performed during the LAVD implantation are displayed in Table 2. Of particular interest is the concomitant left ventricular aneurysmectomy in a patient with a large calcified apical aneurysm and LVAD implantation after a previously performed Dor reconstruction of the left ventricle [17]. Post-implantation, patients stayed in the ICU for an average of 11 ± 10 days and in the cardiology ward for 31 ± 16 days. The in-hospital mortality was 25 %. Two patients died of cerebral haemorrhage; one patient died from sepsis and one patient died from persistent multi-organ failure. In the postoperative phase, 6 (38 %) patients required CVVH for kidney failure and 2 (13 %) patients required ECMO because of severe right ventricular failure. Three (19 %) patients underwent re-thoracotomy because of cardiac tamponade. Two of the patients who died required CVVH, ECMO and re-thoracotomy because of cardiac tamponade. Patients were discharged on average 42 ± 21 days after LVAD implantation.

**Table 2:** Concomitant surgical procedure (*n* = 16)

LV aneurysmectomy	2 (13 %)
TV annuloplasty	13 (81 %)
LAA exclusion	8 (50 %)
AVR	1 (6 %)
PFO closure	1 (6 %)

*AVR* aortic valve replacement; *LAA* left atrial appendix; *PFO* patent foramen ovale; *TV* tricuspid valve

### Adverse events after hospital discharge

After hospital discharge, the readmission rate for clinical adverse events was 44 %. One patient (6 %) had an ischaemic stroke with central facial palsy at day 61 and 2 patients (13 %) were re-admitted for driveline infection, which was treated successfully in both cases with antibiotics. One patient (6 %) was re-admitted to the ICU because of acute renal failure caused by pump thrombosis. This patient was treated successfully with thrombolysis, resulting in good recovery of the LVAD and renal function. None of the patients experienced pump failure, although 1 patient (6 %) had to exchange the controller due to a malfunctioning plug. This controller exchange was successful and the patient encountered no adverse effects.

### Clinical follow-up

**Fig. 2:**
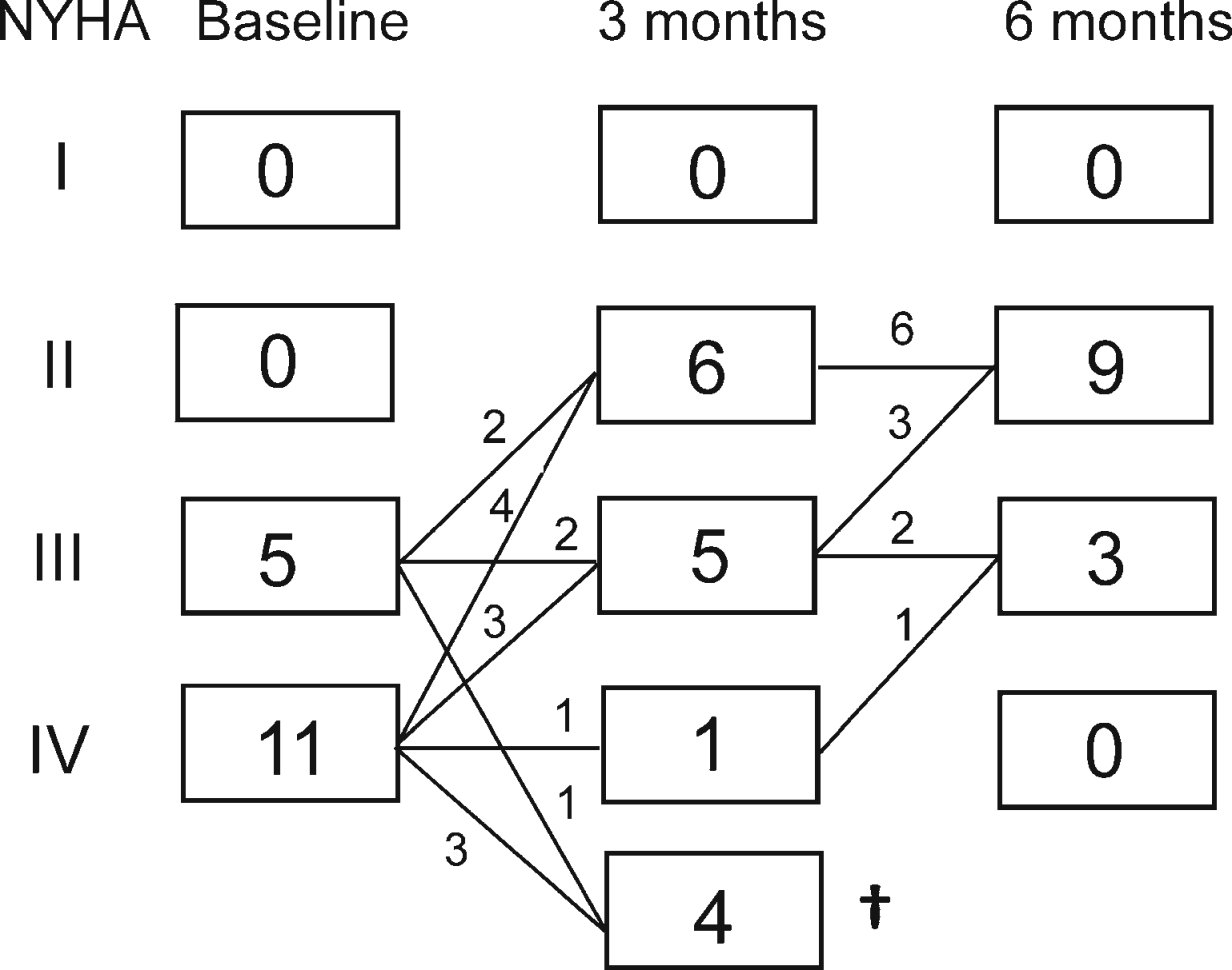
Individual New York Heart Association (NYHA) functional class at baseline, 3 and 6 months follow-up. Mean NYHA functional class improved significantly from 3.7 ± 0.1 pre-implantation to 2.6 ± 0.2 at 3 and 2.3 ± 0.1 at 6 months (*P* < 0.001)

**Fig. 3:**
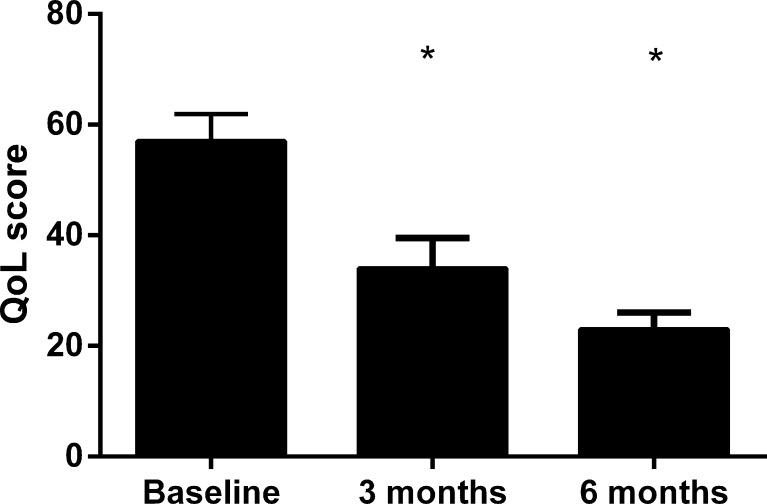
Quality of life, as assessed by the Minnesota Living with Heart Failure questionnaire, demonstrated significant improvement at 3- and 6- month follow-up compared with baseline. Data are presented as mean ± standard error of the mean *P < 0.001 compared with baseline

**Fig. 4:**
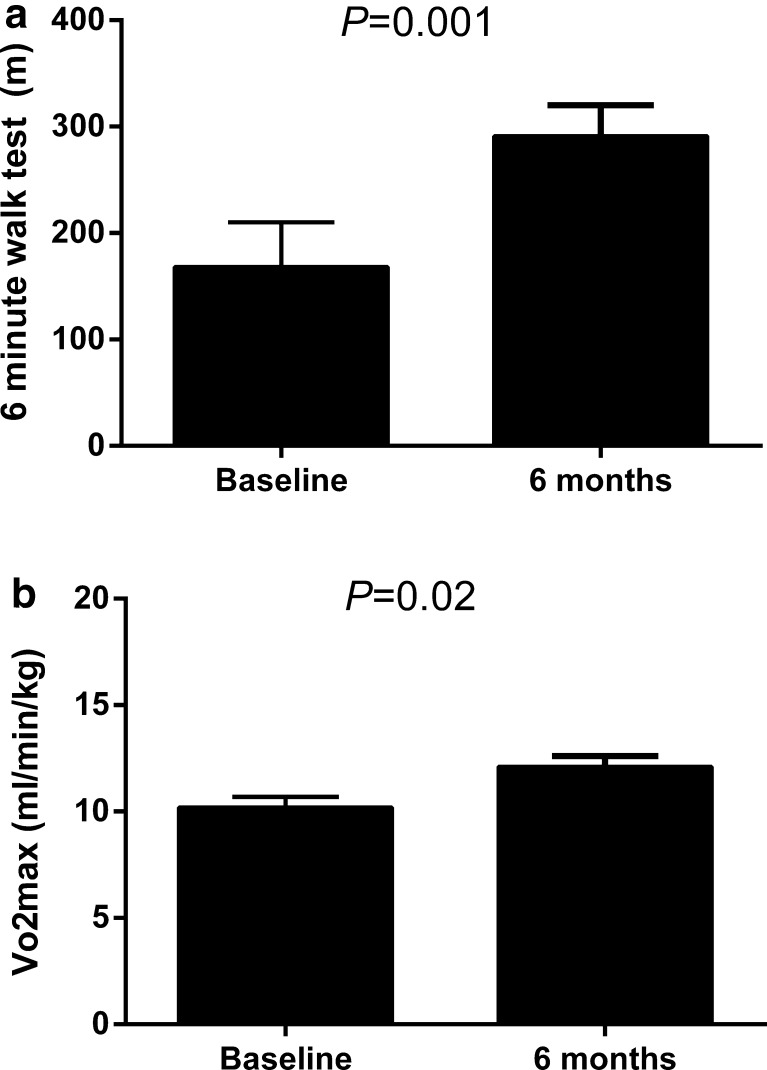
Exercise capacity at baseline and at 6-month follow-up. Panel A shows a significant increase in 6 min walking distance. Panel B shows an improvement in peak oxygen consumption. Data are presented as mean ± standard error of the mean

At 30-day and 6-month follow-up the survival rate was 88 and 75 %, respectively. The NYHA functional class significantly improved from 3.7 ± 0.1 at baseline to 2.6 ± 0.2 at 3 and 2.3 ± 0.1 at 6 months (*P* < 0.001). The individual data are shown in Fig. 2. The improvement in functional class was accompanied by an improvement in quality-of-life (Fig. 3; *P* < 0.001). As shown in Fig. 4, 6 min walking distance and VO_2_ max improved at 6 months (*P* < 0.05). Laboratory tests revealed stable haemoglobin and renal function over time (Table 3). There was a trend towards a decrease in NT-proBNP from 5578 ± 1260 ng/l at baseline, to 3551 ± 942 ng/l at 3 and 3230 ± 649 ng/l at 6 months (*P* = 0.09).

**Table 3:** Laboratory tests (*n* = 16)

	Baseline	3 months	6 months	*P*
Haemoglobin (mmol/l)	7.8 ± 0.2	7.3 ± 0.3	7.5 ± 0.4	0.14
eGFR (ml/min/1.73 m^2^)	54 ± 5	60 ± 5	57 ± 5	0.22
NT-ProBNP (ng/l)	5578 ± 1260	3551 ± 942	3230 ± 649	0.09

*eGFR* estimated glomerular filtration rate; *pro-BNP* N-terminal pro brain natriuretic peptide

## Discussion

The present study evaluates the first LVAD destination program in the Netherlands for end-stage heart failure patients. The main findings are a 6-month survival rate of 75 % and improvement of both functional capacity and quality-of-life during follow-up. These results demonstrate that continuous-flow LVAD therapy is a promising therapeutic option for patients with end-stage heart failure ineligible for heart transplantation.

### Survival

The gold standard for treatment of end-stage heart failure is heart transplant. Survival after transplant is good with reported 1-year survival rates of 85–90 % [18, 19]. However, due to increasing numbers of heart failure patients and scarcity of heart donors, this treatment is limited to a small number of patients. The introduction of the LVAD as bridge to transplant allows better survival on waiting lists and preservation of end-organ function. With improvements in the design of the LVAD, the devices have become more durable and long-term treatment with LVADs is now possible. Recent data demonstrated that current treatment with LVAD results in nearly the same outcome as patients undergoing heart transplant [5, 7, 20]. The fifth INTERMACS report showed that actuarial 1- and 2-year survival for continuous-flow LVADS (all indications, *n* = 5436) is 80 and 70 %, respectively [7]. In the current study survival at 6 months was 75 %. All patients who died developed severe renal failure requiring CCVH and 2 patients also developed right ventricular failure requiring ECMO. In line with this, observed risk factors for early mortality in the INTERMACS report were severe renal dysfunction and severe right ventricular failure. Furthermore, INTERMACS level 1 and 2 are also associated with increased mortality after LVAD implantation [7, 14]. Two of the patients who died in the current study deteriorated from level 2 and 3 at the time of acceptance to level 1 at the time of implantation. These events led to a change in the protocol: a second approval moment was introduced just before implantation. Importantly, it has been noted that patients receiving LVAD as destination therapy carry a higher risk of death than bridge to transplant patients [7]. In particular, irreversible pulmonary hypertension and an impaired renal function make patients more vulnerable to postoperative complications after LVAD implantation. Furthermore, additional complex surgical interventions during implantation may complicate the operation and can affect survival. All patients in the current study were deemed suitable for LVAD as destination therapy and the majority required additional surgical interventions during implantation underlining the complexity and severity of illness in the current patient group.

### Adverse events

Adverse events remain of concern in the treatment of patients with LVAD. The most commonly reported events are infections, right ventricular failure, device failure and thromboembolic events [7, 8, 11, 20]. In the current study, adverse events observed after discharge were driveline infection, pump thrombosis, ischaemic stroke and a malfunctioning plug that required controller exchange. Importantly, renal function and haemoglobin levels remained stable over time and no clinically relevant haemolysis was observed.

Right ventricular failure is an important cause of death in LVAD patients and has been associated with other adverse events such as bleeding and renal failure. The occurrence of right ventricular failure in patients receiving LVAD therapy is approximately 20–35 % [21, 22]. However, in the current study only 2 patients required ECMO because of right ventricular failure. These patients also developed severe renal failure.

Another concern is increased risk of infection. Although pump pocket infections are rare, driveline infections frequently occur and are potentially serious adverse events. The majority of infections develop early after LVAD implantation; however, the risk remains and increases with the duration of the LVAD use [6, 22]. In the current study, 2 patients were re-hospitalised because of driveline infections. Although they were treated successfully, they require chronic antibiotic therapy. The frequency of LVAD-related infections underscores the need for special attention to driveline care and infection prevention. Several studies have demonstrated that the selection process influences outcome [23, 24]. However, with the diversity in LVAD types and indications, comparison and implementation of selection criteria remains challenging. A careful screening program should therefore be implemented with a comprehensive assessment of indications and contraindications.

### Functional capacity and quality-of-life

There was significant improvement in functional capacity and quality-of-life over 6 months. At 6-month follow-up there were no patients in NYHA class IV while the majority were in NYHA class II. Patients reported that they were able to participate in normal daily activities. This was also demonstrated by the significant improvement in quality-of-life. In line with this, several studies demonstrated improvement in functional capacity and quality-of-life after LVAD implantation, despite the occurrence of adverse events [5, 7, 11]. For instance, the Dutch study reported by Pruijsten et al. demonstrated significant functional and haemodynamic improvement after LVAD implantation justifying the use of an LVAD as an alternative to transplant [5]. Further improvement in the design of the LVAD may lead to a reduction of adverse events and further improved quality-of-life.

## Limitations

Our population consisted of the first patients treated with LVAD as destination therapy in the Netherlands. This meant we were faced with new problems that were specific for this patient group and led to changes in our protocol. It should therefore be noted that a learning curve was present throughout the study. The number of patients in the current study is small and follow-up is limited. Further research with a larger study population and longer follow-up will be needed to determine the safety and efficacy of LVADs as destination therapy in end-stage heart failure patients.

## Conclusion

Continuous-flow LVAD therapy is a promising therapeutic option for patients with end-stage heart failure ineligible for heart transplant.
